# Epidemiology of respiratory viruses in patients with severe acute respiratory infections and influenza‐like illness in Suriname

**DOI:** 10.1111/irv.12791

**Published:** 2020-09-02

**Authors:** Meritha Grunberg, Rachel Sno, Malti R. Adhin

**Affiliations:** ^1^ Prof. Dr. Paul C. Flu” Institute for Biomedical Sciences Paramaribo Suriname; ^2^ Department of Biochemistry Faculty of Medical Sciences Anton de Kom Universiteit van Suriname Paramaribo Suriname

**Keywords:** influenza, influenza‐like illness, respiratory syncytial virus, respiratory viruses, seasonality, severe acute respiratory infection, South America, tropical

## Abstract

**Background:**

Influenza has been well studied in developed countries with temperate climates, in contrast to low‐ and middle‐income (LMIC) countries, thus hampering the effort to attain representative global data. Furthermore, data on non‐influenza respiratory infections are also limited. Insight in viral respiratory infections in Suriname, a tropical LMIC in South America, would contribute to improved local preventive measures and a better global understanding of respiratory viruses.

**Methods:**

From May 2016 through April 2018, all patients (n = 1096) enrolled in the national severe acute respiratory infection and influenza‐like illness surveillance were screened for the presence of 10 respiratory viruses with singleplex RT‐PCR.

**Results:**

The overall viral‐positive detection rate was 45.3%, specified as RSV (19.4%), influenza (15.5%), hMPV (4.9%), AdV (4.6%), and parainfluenza (3.8%). Co‐infections were detected in 6.2% of the positive cases. Lower overall positivity was observed in the SARI vs ILI surveillance and influenza prevalence was higher in outpatients (45.0% vs 6.7%), while RSV exhibited the reverse (4.8% vs 23.8%). Respiratory infections in general were more common in children than in adults (54.4% vs 29.5%), although children were significantly less affected by influenza (11.5% vs 22.7%). None of the respiratory viruses displayed a clear seasonal pattern, and viral interference was observed between RSV and influenza.

**Conclusions:**

The comprehensive information presented for Suriname, including first data on non‐influenza respiratory viruses, displayed distinct differences between the viruses, in seasonality, within age groups and between SARI/ILI, accentuating the need, especially for tropical LMIC countries to continue ongoing surveillance and accumulate local data.

## 1 INTRODUCTION

Respiratory infections are the most common infections in humans and are a leading cause of morbidity and mortality throughout the world, especially in developing countries.

The highest burden of disease is among children under the age of 5 years, elderly and subjects with underlying medical conditions and the most common causative agents of respiratory infections are respiratory viruses, such as influenza, human respiratory syncytial virus (RSV), adenovirus (AdV), human metapneumovirus (hMPV), coronavirus (CoV), parainfluenza virus (PIV), and human rhinovirus (HRV).

The epidemiology of influenza, generally characterized as “the flu,” has been well studied in developed countries with temperate climates, but the influenza pandemic in 2009 highlighted the importance of additional data from tropical low‐ and middle‐income countries (LMIC), to attain comprehensive and representative global data. Suriname has earlier conducted a post‐pandemic 1‐year study providing first insight into the influenza dynamics and characteristics.^1^

The World Health Organization (WHO) Global Influenza Surveillance and Response System has been empowering countries to actively, accurately, and efficiently diagnose and respond to influenza viruses of public health concern.^2^

Anno 2019, influenza data are regularly reported across the five continents, while information on the epidemiology, seasonality, and burden of disease of non‐influenza etiological agents of respiratory viral infections (RVI) is still limited. Distressingly, this issue is especially paramount in LMIC, where RVI have the highest socioeconomic impact. Increasing recognition of the importance of other respiratory viruses (ORVs) to the disease burden of acute respiratory illnesses has prompted many countries to supplement their influenza surveillance platforms with ORV diagnostics.

ORVs usually present with influenza‐like non‐specific symptoms as cough and fever, but exhibit distinct differences in their epidemiology, severity of disease, age distribution, and seasonality. RSV is considered as the main etiologic agent for RVI in children under the age of one, often resulting in hospitalizations due to severe lower respiratory tract infections.^3^ hMPV infections display symptoms and sequelae similar to RSV, while HRV is generally associated with mild upper respiratory tract infections.^4^, ^5^ AdV and PIV target both children and adults. PIV consist of four serotypes, but PIV4 is often disregarded within surveillances, due to the usually mild and subclinical course of disease.^6^

In addition, respiratory viruses can co‐exist, allowing multiple infections in one patient, sometimes exacerbating the clinical manifestations, especially in children.

In 2015, Suriname was selected as one of the tropical countries, to receive aid from the WHO’s Pandemic Influenza Preparedness (PIP) Framework. This framework aims to improve global pandemic influenza preparedness and response, through provision of financial and technical support. With this support, Suriname has strengthened the country's capacity to detect and monitor other respiratory viruses and has implemented an expanded severe acute respiratory infection (SARI) surveillance. Since August 2015, data from Suriname are shared directly with Pan American Health Organization and WHO’s global data reporting platforms, FluID and FluNet.

The aim of this study was to gain insight in the epidemiology, distribution, and seasonality of respiratory viruses in Suriname. Etiological agents responsible for RVI were identified in patients, enrolled in the national SARI/ILI surveillance in a 2‐year period from May 2016 through April 2018. This information is not only essential for local prevention and control of RVI, but will also contribute to a better understanding of the global burden of respiratory viruses.

## 2 MATERIALS AND METHODS

### 2.1 Study setting

Suriname is a small tropical country located in the Northern Hemisphere close to the equator, situated along the Northeastern Coast of South America, bordering Guyana to the west, French Guiana to the east, and Brazil to the south. Suriname has a population of just over 575 000, with the majority living in and around the capital Paramaribo.

Respiratory cases are captured within a national SARI and ILI surveillance system. Both systems adhere to the inclusion criteria for acute respiratory infection set by the WHO, fever of ≥38°C and cough with an onset within the last 10 days. SARI cases additionally require hospitalization. The SARI surveillance is performed at three sentinel sites: ‘s Lands Hospital (LH), the intensive care unit and the pediatric ward of the Academic Hospital Paramaribo (AZP), and the rural hospital Mungra Medisch Centrum (MMC) in the border district Nickerie. The national ILI surveillance consisted of a private medical clinic in the capital.

### 2.2 Study population

In the period from May 2016 through April 2018, clinical and demographic information was collected from all patients enrolled in the national surveillance system, meeting the inclusion criteria (n = 1096). Nasopharyngeal/oropharyngeal swabs were tested for the presence of influenza, RSV, hMPV, AdV, and PIV types 1, 2, and 3.

HRV is still not generally included in the national respiratory surveillance. In order to gain some insight in the common cold in Suriname, all samples (n = 593) from a 1‐year period (May 2016 through April 2017) were also screened for HRV presence and these results are presented separately.

### 2.3 Laboratory analysis

Viral RNA was extracted using the QIAamp Viral RNA kit (Qiagen) according to manufacturer's protocol. Presence of influenza, RSV, hMPV, AdV, PIV types 1, 2, and 3, and HRV was detected using singleplex RT‐PCR (StepOnePlus, Applied Biosystems) with AgPath‐ID One‐Step RT‐PCR Reagents (Ambion⁄Applied Biosystems, Life Technologies Corporation, Carlsbad, CA, USA), 6‐carboxyfluorescein (FAM) as reporter and Blackhole Quencher (BHQ) according to earlier protocols.^7^, ^8^

Primers and probes for influenza were obtained from CDC International Reagent Resource (CDC‐IRR). RNase P was used as an internal control.^7^ Additional RT‐PCR testing was conducted to differentiate between influenza A subtypes A(H1N1)pdm09 and A(H3N2), and to distinguish between influenza B Yamagata and Victoria lineage. A(H3N2)‐positive samples were screened for possible pandemic origin (H3N2v).

### 2.4 Data analysis

Data were analyzed with Tableau version 2019.2.2. In the comparative analysis, results on PIV types 1, 2, and 3 are presented as combined PIV result, due to limited individual numbers. The chi‐square test was used for comparisons between the distribution of respiratory viruses within the different groups (gender, ILI, SARI). A *P*‐value < .05 was considered statistically significant. The study population was divided in different age groups: <6 months, 6‐23 months, 2‐4 years, 5‐17 years, 18‐64 years, and ≥65 years, and correlations between viral prevalence and age were analyzed with Pearson's correlation coefficient.

### 2.5 Ethical considerations

Samples and data were collected according to the national regulations for the routine public health surveillance and were therefore not subject to additional requirements of written consent.

## 3 RESULTS

All 1096 samples collected within the study period were successfully processed. The demographic data and the distribution of samples from the sentinel sites with the corresponding positive detection rate (PDR) are presented in Table 1.

**Table 1:** Demographic characteristics and prevalence of viral respiratory infections

Characteristic	Cases	PDR^a^
**Gender**		
Male	608	43.9%
Female	486	46.9%
unknown	2	100.0%
**Age group**		
<6 mo	243	54.3%
6‐23 mo	218	56.4%
2‐4 y	127	54.3%
5‐17 y	110	50.9%
18‐64 y	325	30.8%
≥65 y	72	23.6%
unknown	1	0.0%
**Surveillance system**		
ILI	251	61.4%
SARI	845	40.6%

Abbreviations: ILI, Influenza‐like illness; PDR, Positive detection rate; SARI, severe acute respiratory infection.

^a^ Human Rhinovirus results not included

The male‐to‐female ratio was 1.25, and the majority of the study population consisted of children (63.7%). The median age of the patients was 3 years [range 6 days‐92 years].

LH houses the national bureau of mother and child care in Suriname, which may account for the high percentage of children younger than 5 years old (88.2%) enrolled within this sentinel site.

In the ILI surveillance, children in the age groups <6 months and patients ≥65 years were hardly represented.

**Figure 1:**
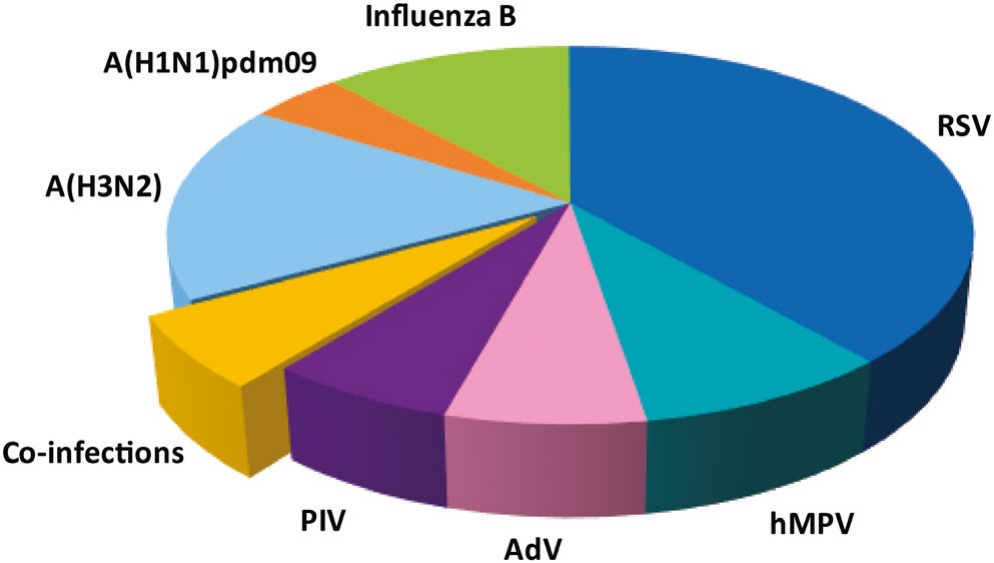
Respiratory virus distribution in Suriname

Respiratory viruses were detected in 45.3% of the samples. With the inclusion of HRV, the overall PDR was elevated to 52.1%. An overview of the PDR for each virus is provided in Table 2A, and the virus distribution within the positive cases is illustrated in Figure 1.

**Table 2:** Overview of virus‐specific prevalence

A			B		
**Respiratory Virus**	**n**	**PDR (%)**	**Co‐infections**	**n**	**PDR** ^a^ **(%)**
Influenza A	109	10.0%	**Double infection**		
*A(H3N2)*	*86*	*7.9%*	RSV‐AdV	10	2.0%
*A(H1N1)pdm09*	*23*	*2.1%*	RSV‐PIV	5	1.0%
Influenza B	61	5.6%	RSV‐hMPV	3	0.6%
Total Influenza ( A & B)	170	15.5%	AdV‐hMPV	3	0.6%
			AdV‐PIV	3	0.6%
RSV	213	19.4%	RSV‐A(H3N2)	1	0.2%
hMPV	54	4.9%	RSV‐Influenza B	1	0.2%
AdV	50	4.6%	hMPV‐PIV	1	0.2%
PIV	42	3.8%	hMPV‐A(H3N2)	1	0.2%
*PIV1*	*18*	*1.6%*	hMPV‐Influenza B	1	0.2%
*PIV2*	*5*	*0.5%*	Influenza B‐PIV	1	0.2%
*PIV3*	*19*	*1.7%*	**Triple infection**		
Total ORV	359	32.8%	A(H1N1)pdm09‐RSV‐hMPV	1	0.2%
			**Total co‐infections**	31	6.2%

Abbreviations: AdV, human adenovirus; hMPV, human metapneumovirus; PDR, Positive detection rate; PIV, parainfluenza virus; RSV, respiratory syncytial virus.

^a^ PDR within positive cases (n = 497, not including human rhinovirus results).

RSV was the most common etiological agent for RVI (19.4%), followed by influenza viruses (15.5%). The majority of the influenza‐positive patients harbored A(H3N2) (50.6%), followed by influenza B virus (35.9%), while only 13.5% was caused by influenza A(H1N1)pdm09. Determination of possible pandemic origin (H3N2v) of all A(H3N2)‐positive samples yielded no A(H3N2) variants. Influenza B lineage determination displayed the presence of both Victoria (72.1%) and Yamagata lineage (23.0%). Lineage determination was unsuccessful for three weak B‐positive cases.

Co‐infections were detected in 6.2% of the positive cases. The frequency of occurrence of the combinations of these co‐infections is shown in Table 2B. Co‐infections of two influenza viruses were not observed, while 1.2% of the positive patients harbored mixed infections of influenza with non‐influenza viruses. Co‐infections of two ORVs predominated, but were only registered in children and mostly in the age group of 6‐23 months. Influenza had the lowest tendency of occurring with another respiratory virus (3.5%), followed by RSV (9.9%), while PIV2 was predominantly seen as co‐infection (80.0%). Adenovirus co‐infections were also quite common (32.0%), but interestingly, no co‐infections of influenza and AdV were observed. Inclusion of HRV results revealed 8 additional co‐infections, including 5 co‐infections with AdV, 2 co‐infections with RSV, and 1 triple infection with both AdV and RSV.

### 3.1 Gender and Age distribution

No significant difference was detected between males and females with respect to influenza infections (*P* = .943), while RSV was significantly more prevalent in females under 5 years old (*P* < .001). Similarly, a higher HRV presence was observed in females than males, although in adults. Low numbers for HRV positives may have distorted this observation.

**Figure 2:**
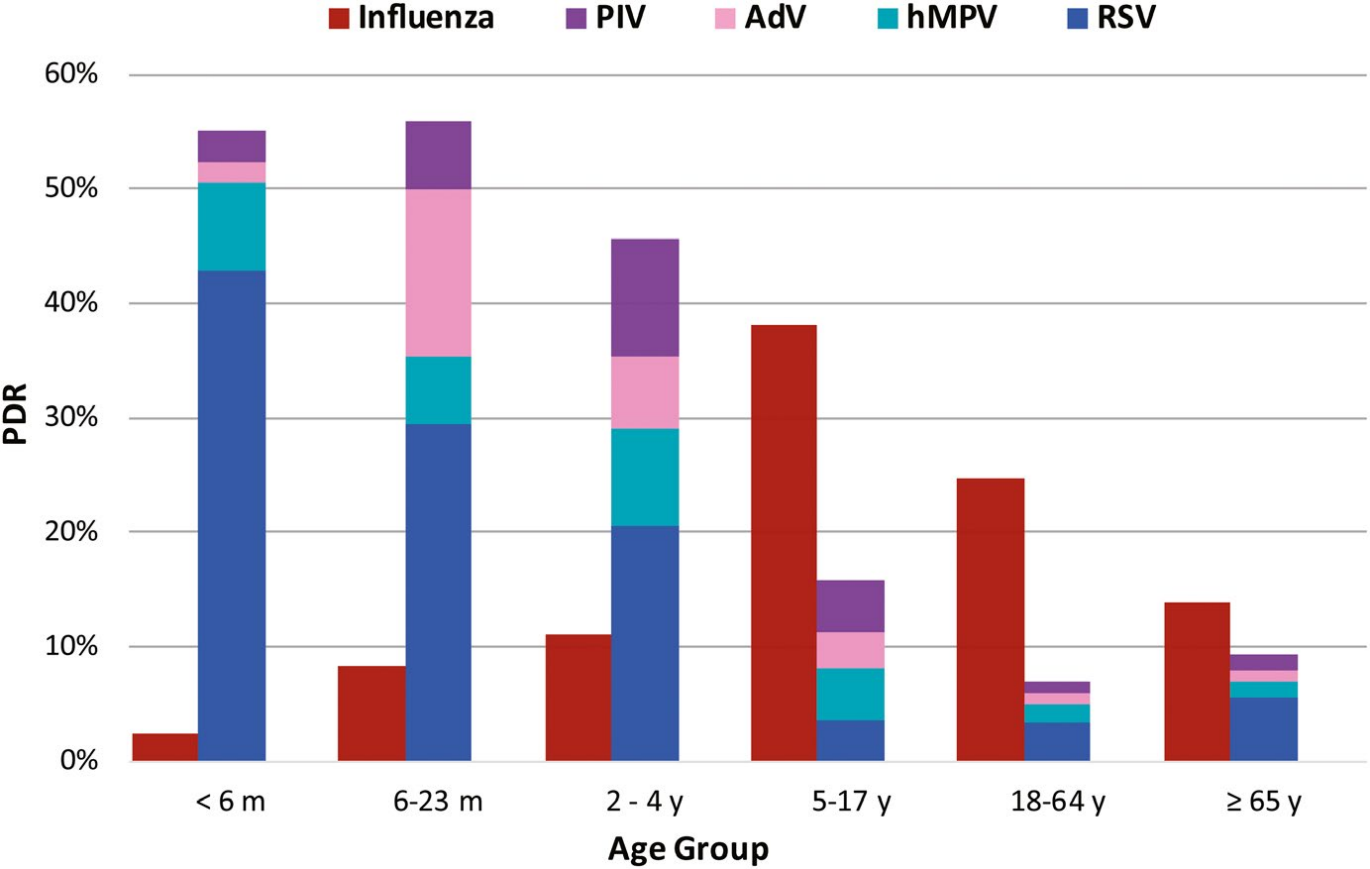
Prevalence of Influenza and ORV per age group

Within the age groups, distinct differences were noted in the overall viral positivity (Table 1) and an overview of virus prevalence per age group is depicted in Figure 2.

Respiratory infections in general were more common in children than in adults (54.4% vs 29.5%, *P* < .001). However, adults were significantly more affected by influenza than children (22.7% vs 11.5%, *P* < .001), as was reflected in a median age of 19 years ranging from 9 years for influenza B to 32 years for influenza A(H1N1)pdm09.

In addition, within the category of children, a positive correlation (*r* = 0.98) was revealed between the overall occurrence of influenza and age. On the other hand, non‐influenza respiratory viruses were mostly present in children (Figure 2), with the highest occurrence in children under the age of 5 years, as reflected in a median age of 8 months ranging from 6 months for RSV to 2 years for PIV. RSV was the predominant virus in children under the age of 5 years, with the highest occurrence in infants under 6 months (42.8%), while the presence generally declined with the progression of age (*r *= −0.91).

HRV was also prominent in infants under 6 months (13.2%), while AdV and PIV were more frequently registered in children under the age of 5 years. hMPV occurrence was similar in all age groups of children.

### 3.2 ILI vs SARI

The majority of cases originated from SARI sentinel sites (Table 1), but the overall PDR was significantly lower than in the ILI surveillance (*P* < .001). Influenza prevalence was distinctly higher in outpatients (45.0% vs 6.7%; *P* < .001), while the reverse was observed for RSV (4.8% vs 23.8%; *P* < .001). HRV was predominantly circulating in hospitalized patients and was only detected in outpatients in the age group 18‐64 years. No significant differences were detected for the other viruses. It should be noted that the distribution of different age groups in the ILI and SARI surveillance was skewed (data not shown), complicating comparisons.

### 3.3 Seasonal distribution

**Figure 3:**
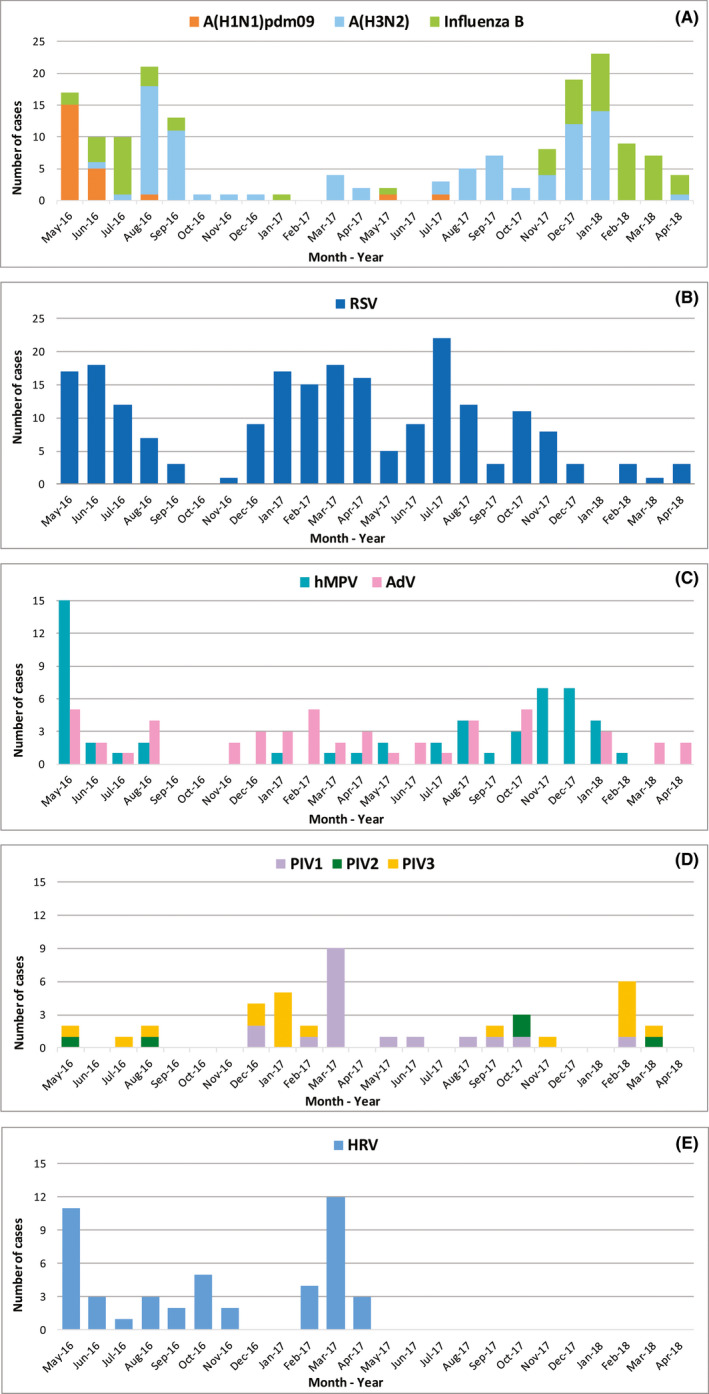
Seasonal distribution of respiratory viruses in Suriname

The monthly number of positive cases for each respiratory virus is illustrated in Figure 3. None of the respiratory viruses displayed a clear seasonal pattern. The study period started with a relatively high activity of respiratory illness with simultaneous circulation of influenza viruses and various ORVs. This period lasted 4 months, after which especially ORV activity tapered off. Peak activity for influenza was registered in January 2018, with A(H3N2) as main agent. Influenza A(H1N1)pdm09 activity was, with the exception of the first 2 months, quite limited throughout the whole period. Influenza viruses displayed discrete periods of peak activity in Suriname, not corresponding with fixed periods throughout the year (Figure 3A).

RSV and AdV exhibited an almost constant presence in Suriname throughout the entire study period (Figure 3B,C), although AdV displayed a considerably lower prevalence.

Periods of relatively discrete activity, without a clear seasonal pattern, were observed for hMPV (Figure 3C) and PIV (Figure 3D). Interestingly, PIV2 was only observed as co‐circulating PIV, although the low numbers prohibited clear conclusions.

HRV presence was detected throughout the study period, except for December and January. Nonetheless, results for just 1 year excluded statements about seasonal HRV trends.

**Figure 4:**
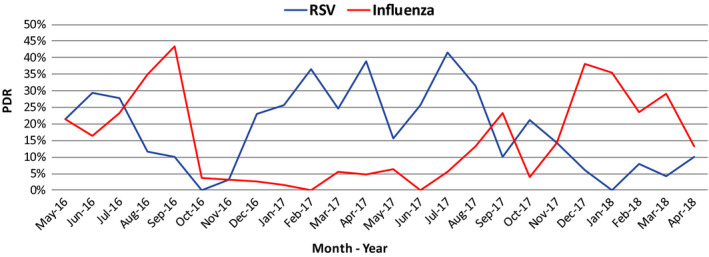
Viral Interference between RSV and Influenza

Also noteworthy, was the finding of an inverse correlation between the occurrence of RSV and influenza as illustrated in Figure 4, where RSV displayed reduced activity in periods with relatively high influenza circulation.

## 4 DISCUSSION

Most studies on respiratory infections are either focused on key populations (children, elderly) or include just SARI or ILI data, but we addressed epidemiological patterns of both influenza and ORVs within the ILI and SARI patients in Suriname without age restrictions. Data reported on prevalence is influenced by a variety of factors, including respiratory viruses tested, the study period, the target population, SARI or ILI cases, severity of the epidemic, the geographic location, the climate, and the historic disease burden of the area, evidenced by the heterogeneity of international data. Therefore, data comparison with different studies proved to be a challenging process. The overall PDR of 45.3% was comparable to studies done in South Arizona and Bolivia, although both surveys tested for additional viruses.^9^, ^10^ In addition, the findings for Suriname revealed a higher PDR than Ecuador (45.3% vs 26.8%), despite the similarities as tropical South American countries and the use of matching respiratory viruses and an analogous population in terms of age distribution.^11^ However, their study was conducted in another period and ORVs were detected with the less sensitive direct immunofluorescence assay, which may account for the diverging PDR results.

The finding of RSV and influenza as leading respiratory viruses was consistent with studies in Thailand and Ecuador, but in contrast with results from Gabon, where RSV and influenza were preceded by AdV and PIV.^11^, ^12^, ^13^ The actual RSV burden could still be underestimated, because the customary ILI/SARI inclusion criteria are not optimal for particular identification of RSV cases, which often present without fever.

The overall PDR in Suriname for the other tested viral pathogens AdV, PIV, and hMPV displayed a range from 3.8% for PIV to 4.9% for hMPV and although data on prevalence of respiratory viruses other than influenza and RSV are limited, basic comparison with analogous country settings revealed similar PDRs for PIV and AdV in Brazil and some Central American countries.^14^, ^15^ Interestingly, hMPV had the leading role in our setting for the three viruses, which not only diverged from Brazil, with equivalent PDRs for these three viruses, but was in contrast with Central American countries, where hMPV ranked at the bottom.^14^, ^15^

This finding gained in importance, considering the high occurrence of RSV in our population, as it has been shown that RSV infection can also induce cross‐protection against hMPV, thus even negatively affecting the epidemiological pattern of this respiratory pathogen in Suriname.^16^ On the other hand, the global presence of hMPV, AdV, and PIV seems to be variable without a particular order of occurrence for these viruses.

All three investigated PIV types were detected in our study, with a predominance of PIV3, similar to other studies.^6^, ^17^

The detected PDR of 7.8% for HRV in Suriname was almost identical to reports from Bolivia and Gabon.^10^, ^12^

Simultaneous infection with more than one respiratory virus was demonstrated in 6.2% of the positive cases, almost exclusively in children, corroborating earlier studies describing co‐infections as a common phenomenon in pediatric populations.^17^

We revealed that adenoviruses were prone to coexistence, since 32.0% of the AdV‐positive cases also harbored another respiratory virus as co‐pathogen. This conclusion gained in strength with the addition of the limited HRV data resulting in a 42.0% co‐infection rate. Similar observations about co‐infections with AdV were reported in the Eastern Mediterranean region and in Gabon.^12^, ^17^ Our findings add to the data of high AdV occurrence in co‐infections and linked to the high prevalence of asymptomatic AdV infections in children also supported the hypothesis that adenoviruses may not be the cause of respiratory symptoms.^12^

Interestingly, the leading respiratory viruses, RSV and influenza, exhibited the lowest number of co‐infections in Suriname, corroborating findings from Brazil, where co‐infections with another respiratory virus were less common for RSV and influenza A infections.^18^

The observation in Suriname of delayed or less intense RSV peaks during influenza epidemics substantiated the phenomenon of viral interference, a tendency also documented in Ecuador and Hong Kong.^11^, ^19^ The exact mechanism of viral interference is still unknown, but it has been postulated that prolonged shedding from the primary virus infection could provide temporary immune protection against infection by another respiratory virus, but an existing competition of these two viruses for the same ecological niche could also account for the low co‐infection rate and the observed displacement between these two viruses.^20^, ^21^

The absence of significant differences in the influenza detection between males and females was consistent with previous studies.^11^, ^17^

Gender was also not a risk factor for the non‐influenza respiratory infections, except for RSV and HRV. The significantly higher presence of RSV noted in this study in females under 5 years old was in contrast with earlier reported male predominance.^22^, ^23^ The global data regarding gender‐related susceptibility to RSV infection are ambiguous, and our results added to the view that notwithstanding clear immunological differences between the sexes, the relationship between sex and risk of respiratory infections remains controversial.

In concordance with global data, influenza morbidity rates varied among patients from different age groups and young children were the least likely to be positive for influenza.^11^, ^17^ Differences in median age and morbidity rates are generally impacted by study population, study period, immune status, risk of exposure, and health system, but it should be noted that the numbers from Suriname were also influenced by the lack of influenza vaccination of children and elderly persons.

In contrast to influenza, all investigated non‐influenza respiratory pathogens predominated in children under 5 years old, consistent with earlier published data and the respective age group distribution was also in line with general observations for non‐influenza viruses.^11^, ^17^ RSV was mainly detected in infants under 6 months with a PDR of 42.8%, emphasizing the generally accepted leading role of RSV in respiratory infections in children.

AdV does not seem to be an important contributor to respiratory disease in adults, as less than 1% in the adult population in this study was affected, consistent with findings from a Caribbean study.^24^

The HRV prevalence observed in this study in children younger than 6 months provided support for the findings from Latin American countries that HRV predominately targets children less than 1 year old.^25^

Scarce data are available for ILI and SARI information within one study population, especially for South America, thereby restricting the options for data comparison. The presentation of ILI and SARI data from Suriname is therefore a valuable addition to the international body of knowledge, particularly for the non‐influenza respiratory viruses. Comparative analysis with a study from Thailand revealed analogous findings as a lower overall PDR in the SARI vs the ILI surveillance and the absence of significant PDR differences between both systems for all non‐influenza respiratory viruses, except RSV.^13^ Considering the fact that all samples were collected from symptomatic cases, the lower ratio of positive SARI patients could reflect a higher diversity of etiological causes for respiratory illness in this hospitalized group. Furthermore, reduced influenza prevalence was demonstrated in the SARI surveillance, while the reverse was observed for RSV, which could be partly attributed to the dissimilar age distribution between the groups. The greater representation of children within the SARI surveillance was linked to the participating pediatric hospital wards, while underrepresentation of the age groups <6 months and ≥65 years in the ILI surveillance was probably due to deep‐seated concerns regarding sick infants and elderly persons, resulting in a greater tendency to visit hospital‐associated clinics, which unfortunately are not registered as ILI sentinel sites. However, significant PDR differences between ILI and SARI cases were even registered within the same age group, illustrated by the age group 5‐17 years, with a record influenza PDR in the ILI system (59.0%) vs just 12.2% for this age group in SARI patients (*P* < .001). Various other characteristics of the surveillance systems, including access to care, variations in disease severity and manifestations, may have caused the observed PDR differences within the same age groups.

Expanding the ILI surveillance in general and including hospital‐associated clinics is imperative to achieve more comprehensive representative data.

Data on seasonality are the fundamental tool, aiding in the formulation of recommendations for vaccination timing and vaccine content. Although seasonality data of influenza and RSV are collected from several countries through open‐access online data from WHO and Pan American Health Organization platforms, the information regarding the circulation of other respiratory viruses is still scanty. Even influenza and RSV data from neighboring countries Guyana and French Guiana are limited and seasonal distribution of influenza and RSV in Suriname was compared to other tropical countries in South America and the Caribbean. Suriname is ranked within FluNet in the category of tropical South America (TSA) and is placed in the Caribbean section of SARInet.

As expected, the seasonality of influenza in Suriname was unlike temperate countries, but concurrent with results from our previous study in Suriname and with data from other TSA countries, displaying discrete periods of peak activity, although not corresponding with fixed seasons throughout the year.^1^ The irregularity of the peak timings also supported earlier findings that seasonality is even less clearly defined in tropical countries close to the equator.^26^

The distribution of influenza in Suriname generally followed the documented overall patterns for TSA and Caribbean countries, with two notable exceptions. Early 2018, influenza activity peaked throughout the region, but unlike TSA countries with A(H1N1)pdm09 as predominant circulating influenza type, Suriname exhibited increased A(H3N2) and influenza B activity, while A(H1N1)pdm09 remained undetected. Secondly, a deviation was observed between January to July 2017, with low influenza cases in Suriname, in contrast to peak activity registered in the generalized influenza patterns both for TSA and Caribbean countries. The non‐conformity in this period could not be explained by less sampling, attested by the lack of significant differences in the amount of collected samples, but the high RSV activity probably negatively impacted influenza infections. Furthermore, other countries in the region as Colombia and Peru also displayed a relatively low influenza activity not only in this period, but throughout 2017.

It should be noted that generalized influenza patterns, based on cumulative data for the TSA countries, provide valuable insight for the region, but close examination of influenza activity in individual countries revealed substantial deviations from this overall pattern, not only regarding the timing of peak activity but also the influenza type. The results from Suriname underscored the view that caution is warranted in case of country projections only based on the generalized pattern, which is particularly evident for small countries with relatively low numbers of cases. Review of their vaccine choice with consideration of national data is therefore a prerequisite to effectively reduce their burden of influenza.

RSV occurrence in Suriname did not exhibit a clear seasonality, in contrast with other tropical countries.^26^

The finding that influenza and RSV occurrence appeared to be inversely correlated added to the evidence for viral interference.

The lack of clear seasonal trends for either AdV, hMPV, or PIV was concurrent with earlier findings, although the number of our reviewed positive cases was limited.^11^ The study had other limitations such as deficiencies in the extent of the national surveillance system. In addition, the SARI surveillance was biased toward the inclusion of children, while the reverse was true for the ILI surveillance, which consisted of only one, although wide‐ranging sentinel site in the capital. Inclusion of additional sentinel sites is highly recommended to augment the representativeness of the national respiratory surveillance data.

The presented results encompassed a period of 2 years, complicating the determination of seasonality, even though it is unlikely that the use of extended study periods will add details to patterns of perennial activity.

Nevertheless, comprehensive information over a 2‐year span has been presented on the viral etiology and seasonality of respiratory infections among both SARI and ILI patients from all age groups, including first data on prevalence, seasonality, and age and gender aspects of non‐influenza respiratory viruses in Suriname, thus supplementing the global data. The results allowed for improved identification of risk groups, enabling Suriname to implement targeted preventive measures and more efficient resource allocation to alleviate the disease burden. The observed divergences from the generalized influenza pattern for TSA countries should encourage Suriname and other tropical countries to accumulate national data.

## CONFLICT OF INTEREST

We do not have any conflict of Interest.

## AUTHOR CONTRIBUTION

**Meritha Grunberg:** Conceptualization (supporting); Data curation (equal); Formal analysis (supporting); Investigation (supporting); Methodology (supporting); Writing‐original draft (supporting); Writing‐review & editing (supporting). **Rachel Sno:** Conceptualization (supporting); Data curation (equal); Formal analysis (supporting); Investigation (supporting); Methodology (supporting); Writing‐original draft (supporting); Writing‐review & editing (supporting). **Malti R. Adhin:** Conceptualization (lead); Formal analysis (lead); Investigation (lead); Methodology (lead); Project administration (lead); Visualization (lead); Writing‐original draft (lead); Writing‐review & editing (lead).
